# Why Arboviruses Can Be Neglected Tropical Diseases

**DOI:** 10.1371/journal.pntd.0000247

**Published:** 2008-06-25

**Authors:** A. Desiree LaBeaud

**Affiliations:** Department of Pediatrics and Center for Global Health and Diseases, Case Western Reserve University, Cleveland, Ohio, United States of America; University of South Florida, United States of America

*PLoS Neglected Tropical Diseases* focuses its scope on the “neglected” communicable diseases of developing countries, diseases that do not receive attention from the developed world. The list of neglected tropical diseases (NTDs) is mainly comprised of helminths, protozoa, and many tropical bacterial species that currently do not receive sufficient priority in international public health programs and research agendas. In a practical sense, these very prevalent diseases of underdevelopment are seen as neglected because they are outside the purview of the Global Fund and its related programs, which concentrate only on HIV, tuberculosis (TB), and malaria. Their neglect occurs despite the fact that NTDs provide an equal share of the global burden of disease, both in terms of chronic disability and mortality. The developed countries' nearly exclusive interest in HIV, TB, and malaria frames the NTD problem, and programs focused only on the “big three”, in many respects, define the neglect of other tropical diseases.

As interest in the NTDs spreads, however, it is important to re-examine the scope of diseases that should be included under the term “neglected tropical disease”. In this Viewpoint article, my premise is that the “neglected tropical viruses”, in particular the arthropod-borne or arboviruses, are a disease group that deserves to be considered among the NTDs. Recently, the World Health Organization added dengue virus as the first arbovirus to its list of NTDs. Because of their link to poverty in the developing world, I believe there are other arboviruses that should also follow suit.

Historically, arboviruses have been most extensively studied by researchers in developed countries. As a result, we know a great deal about their molecular biology, their pathogenesis, and their potential to re-emerge as threats to the developed world. At first glance, arboviruses would seem to be already well represented in the international research agenda, particularly under the rubric of emerging/re-emerging diseases or biodefense pathogens. However, several aspects of arboviral infection and disease consequence have been neglected. Arboviruses affect the impoverished more severely and promote poverty in endemic regions by causing long-lasting sequelae and devastating impacts on quality of life. If NTDs are defined as a group of infectious tropical diseases in developing countries that are both *poverty-promoting* and *long-lasting* in their health impact, then arboviruses certainly qualify as NTDs.

Tropical arboviral infections, like other NTDs, occur in poor urban and rural environments and disproportionately affect low-income populations. As an example, Rift Valley fever virus (RVFV) is embedded in the ecosystems of many poor African nations, but during outbreaks it disproportionately attacks the personal health and family incomes of semi-nomadic pastoralists [1]. This vulnerable group is at risk for RVFV because resource-limited conditions lead to high-risk animal husbandry practices and lifestyle. Other neglected viral infections, such as Japanese encephalitis, dengue, and chikungunya, afflict the poor more frequently and more severely. Arboviral disease outcomes can contribute to poverty, perpetuating a vicious cycle of disease, poverty, and health care injustice.

The recent severe outbreaks of chikungunya virus in India and Africa demonstrate the propensity for arboviral infections to target the poor and exacerbate the problems of poverty. A study of the 2005–2006 chikungunya virus outbreak in India suggested that poor people were more commonly affected and that loss of income while ill led to worsening poverty [2]. Although indicators of poverty are rarely tracked in seroepidemiologic studies of arboviral outbreaks, poor housing and living conditions are usually blamed for disease occurrence [3].

Poverty is an important determinant of arboviral infections, and even within wealthy industrialized nations, foci of arboviral transmission arise in pockets of poverty. For example, in the border counties of Texas where dengue virus infection is common, recent or remote dengue infection has been associated with very low family income (less than US$100/week) [4]. In this fashion, tropical climate and poverty nearly guarantee continued arboviral outbreaks and disease, with disease transmission ecology ignoring political, but not socioeconomic, boundaries.

Poverty is not sufficient, by itself, to promote arboviral outbreaks; however, a community's inability to provide adequate vector control can perpetuate the spread of these diseases. Just as a multi-drug administration can control many of the parasitic NTDs, integrated mosquito control can prevent many of the neglected tropical viral diseases. Unfortunately, those who are impoverished are not able to obtain either the necessary drug therapies or the needed mosquito control, so the NTDs continue to afflict millions of people worldwide. It should be noted that treated bednets, which have proven most effective against malaria transmission, are most active against biting *Anopheles* spp. mosquitoes, which live peridomestically and feed at night. Most mosquitoes carrying arboviruses are not anopheline species. These types of mosquito can feed during the day or at dusk, both outdoors and indoors, meaning that bednets will not be a panacea for all mosquito-borne diseases.

Too often, the environmental conditions around urban slums result in perfect larval development sites that will easily support endemic mosquito cycling of disease [5],[6]. *Aedes aegypti*, the primary vector of many important arboviral diseases, such as yellow fever, dengue, and chikungunya, has been difficult to eradicate and is responsible for the resurgence of many of these diseases [7]. Infections that were once under control, such as yellow fever and Japanese encephalitis, are now capable of returning with a vengeance to poor neighborhoods in both wealthy and impoverished nations [8],[9]. The current outbreaks of yellow fever in Paraguay and Brazil demonstrate our inability to control the resurgence of these arboviral diseases [10]–[12]. Practices to escape poverty are also to blame: the spread of yellow fever in Africa and its reemergence in Brazil has been linked to deforestation [13].

Access to health care is another important issue for the neglected viral diseases. Although many viral infections are acute in nature and often resolve spontaneously, poor patients come to medical care late in the course of disease, as is often seen with dengue infections. This late presentation leads to worsened disease outcomes than for patients who are financially better off and present sooner [14].

Because viral NTDs occur more commonly in those who are poverty-stricken, it can be difficult to decipher all the ways in which the diseases contribute to poverty. Like other NTDs, viral NTDs can lead to debilitating chronic sequelae that impact individual work productivity and family income. RVFV, for example, can lead to permanent visual loss or impairment, which can have a great impact on long-term productivity and quality of life [15],[16]. Importantly, viral NTD outbreaks generally lead to loss of life, family hardship, and distress. Japanese encephalitis is an extreme example, where case fatality rates can be as high as 30%, with one-third of survivors suffering severe neurological sequelae [17]. Because epidemics of arboviruses often receive notice only when they are acute and massive, the public loses sight of ongoing transmission, which has a significant daily impact on the life of people living in endemic countries.

Too often these diseases are ignored and neglected because they have not yet impacted the lives of those living in affluent areas. Many of these diseases are understudied and go unnoticed until outbreaks affect North Americans or Europeans, as recently displayed in the recent surge of interest in chikungunya after outbreaks on the Indian Ocean islands, a favorite European vacation destination [18]. Certain populations within these neglected diseases also go unnoticed, such as the impact chikungunya and dengue have had on neonates and children [19]–[21]. The lack of research contributes to our simplified understanding of these infections and our inability to decipher one infection from another. For example, the understudied Mayaro virus may constitute a significant portion of dengue-like illness cases in Latin America [22].

As more emphasis is placed on defining the true cost of the NTDs, the real-world problem of concurrent and overlapping infections must be re-evaluated [23]. Although the neglected tropical viral diseases are part of poverty in tropical nations, their full impact is likely not to be determined in isolation, as arboviruses constitute a part of the larger health problem in resource-poor settings. More research, more insight, and more discussions regarding these important NTDs and the populations that suffer from them will be necessary to fully define the impact of neglected tropical viruses in the regions where they persist.
